# Supplementary material for: Genetic Characterization of a Sheep Population in Oaxaca, Mexico: The Chocholteca Creole
Source: Animals (Basel). 2021 Apr 20;11(4):1172. doi: 10.3390/ani11041172 (PMC8073038; doi:10.3390/ani11041172)
Supplement: Supplementary file 1 [file animals-11-01172-s001.zip › animals-1107896-SI.pdf]

Supplement 1. Table 4. Parameters of genetic diversity of the 28 breeds. The table shows values corresponding to Ae, Ho He, Fis and Fis IC from each sheep used in the analisys.

	Ae	Ho	He	Fis	Fis IC (95%)
**MEXO**	3.01	0.6783	0.6568	-0.03352	(-0.10490 - -0.00137)
**CChO**	3.327	0.6059	0.6847	0.11703	(0.03578 - 0.15433)
**CHI**	3.22	0.6278	0.6967	0.10050	(0.03575 - 0.13188)
**BR**	2.62	0.5689	0.5604	-0.01433	(-0.09125 - 0.01864)
**COL**	3.29	0.6472	0.6957	0.07079	(0.00435 - 0.10399)
**ECU**	3.03	0.6439	0.6554	0.01820	(-0.07060 - 0.04410)
**PGY**	3.41	0.6751	0.7369	0.08525	(0.01239 - 0.12402)
**ARG**	3.32	0.6070	0.7039	0.13970	(0.07564 - 0.16798)
**BLK**	3.35	0.6399	0.7053	0.09481	(0.02738 - 0.10971)
**PECU**	2.93	0.5311	0.6284	0.15717	(0.07800 - 0.20340)
**PECUB**	3.31	0.6204	0.7024	0.12011	(0.01876 - 0.16116)
**PEMEX**	3.63	0.6165	0.7185	0.14446	(0.08316 - 0.16769)
**PEKAT**	3.04	0.6154	0.6606	0.07112	(-0.03926 - 0.08204)
**PSAL**	2.99	0.6255	0.6385	0.02095	(-0.06595 - 0.06000)
**MALL**	3.22	0.6111	0.6644	0.08149	(0.02005 - 0.11343)
**MEN**	3.41	0.6394	0.7247	0.11949	(0.04444 - 0.16022)
**ORM**	3.39	0.6347	0.7108	0.10868	(0.04534 - 0.14099)
**CHU**	3.50	0.6570	0.7384	0.11201	(0.05036 - 0.13725)
**SEG**	3.48	0.7125	0.7408	0.03889	(-0.01704 - 0.05975)
**CAN**	3.27	0.6484	0.7113	0.08977	(0.02195 - 0.12356)
**PAL**	2.70	0.5764	0.5784	0.00360	(-0.07264 - 0.04326)
**PB**	3.21	0.6309	0.6924	0.09029	(0.01938 - 0.12306)
**ME**	3.22	0.6478	0.7028	0.07946	(0.02347 - 0.10171)
**PCHI**	3.33	0.7002	0.7159	0.02243	(-0.05278 - 0.04584)
**BAL**	2.99	0.6041	0.6384	0.05463	(-0.00385 - 0.07874)
**SI**	3.28	0.6800	0.7086	0.04144	(-0.03117 - 0.05546)

**UDA**	3.17	0.6146	0.6841	0.10318	(0.03661 - 0.13182)
**DORP**	2.73	0.5137	0.6100	0.17653	(-0.10749 - 0.18274)

Ae: number of Allele expected, Ho: observed heterozigosity, He: expected heterozygosity, Fis: coefficient of consanguinity, Fis IC: confidence interval.

Supplement 2. Genetic Distances (DA) are shown in red and the coefficient of genetic differentiation (FST) in blue between pairs of the populations used. The values for Oaxacan Creole are highlighted in black.

	**1**	2	3	4	5	6	7	8	9	10	11	12	13	14	15	16	17	18	19	20	21	22	23	24	25	26	27	28
**CChO**	***	**0.115**	**0.122**	**0.218**	**0.098**	**0.145**	**0.126**	**0.088**	**0.118**	**0.159**	**0.124**	**0.121**	**0.163**	**0.143**	**0.126**	**0.092**	**0.113**	**0.102**	**0.099**	**0.132**	**0.22**	**0.114**	**0.139**	**0.108**	**0.175**	**0.14**	**0.149**	**0.174**
MEXO	**0.125**	***	0.097	0.161	0.091	0.086	0.083	0.073	0.084	0.154	0.115	0.106	0.123	0.124	0.082	0.061	0.088	0.069	0.061	0.106	0.169	0.103	0.089	0.071	0.142	0.097	0.092	0.131
CHI	**0.132**	0.104	***	0.157	0.092	0.091	0.074	0.091	0.081	0.113	0.074	0.081	0.116	0.096	0.087	0.068	0.08	0.065	0.067	0.095	0.155	0.095	0.097	0.079	0.114	0.083	0.099	0.147
BR	**0.247**	0.179	0.173	***	0.133	0.086	0.127	0.164	0.115	0.192	0.166	0.134	0.146	0.108	0.135	0.137	0.146	0.132	0.128	0.132	0.235	0.179	0.121	0.125	0.19	0.155	0.176	0.162
COL	**0.105**	0.098	0.098	0.145	***	0.079	0.086	0.086	0.053	0.112	0.075	0.059	0.093	0.071	0.098	0.075	0.104	0.07	0.065	0.097	0.149	0.058	0.092	0.071	0.105	0.074	0.072	0.083
ECU	**0.157**	0.092	0.097	0.092	0.084	***	0.081	0.094	0.061	0.132	0.109	0.066	0.105	0.042	0.086	0.091	0.091	0.07	0.066	0.082	0.169	0.109	0.084	0.083	0.142	0.093	0.1	0.125
PGY	**0.137**	0.089	0.079	0.139	0.092	0.087	***	0.065	0.087	0.112	0.085	0.071	0.083	0.097	0.087	0.061	0.086	0.054	0.044	0.068	0.146	0.092	0.078	0.067	0.116	0.081	0.093	0.1
ARG	**0.094**	0.078	0.098	0.182	0.092	0.101	0.07	***	0.089	0.119	0.096	0.083	0.109	0.099	0.103	0.049	0.087	0.058	0.057	0.081	0.138	0.096	0.102	0.075	0.145	0.112	0.113	0.113
BLK	**0.128**	0.091	0.088	0.125	0.057	0.066	0.093	0.096	***	0.105	0.052	0.025	0.082	0.053	0.083	0.059	0.075	0.066	0.064	0.093	0.171	0.09	0.079	0.065	0.106	0.073	0.081	0.09
PECU	**0.176**	0.17	0.123	0.215	0.121	0.144	0.122	0.13	0.114	***	0.105	0.108	0.159	0.122	0.111	0.116	0.124	0.107	0.107	0.128	0.21	0.129	0.136	0.12	0.129	0.108	0.135	0.166
PECUB	**0.135**	0.127	0.181	0.184	0.081	0.118	0.093	0.106	0.058	0.115	***	0.044	0.086	0.095	0.092	0.067	0.073	0.07	0.064	0.086	0.144	0.078	0.101	0.077	0.082	0.065	0.084	0.111
PEMEX	**0.131**	0.115	0.087	0.147	0.063	0.072	0.076	0.09	0.177	0.095	0.077	***	0.07	0.065	0.084	0.063	0.069	0.059	0.056	0.075	0.149	0.08	0.097	0.073	0.107	0.069	0.085	0.073
PEKAT	**0.179**	0.135	0.126	0.161	0.1	0.114	0.09	0.119	0.09	0.177	0.095	0.077	***	0.109	0.126	0.101	0.115	0.1	0.087	0.123	0.188	0.118	0.14	0.113	0.135	0.108	0.114	0.116
PSAL	**0.156**	0.135	0.103	0.117	0.076	0.044	0.104	0.107	0.057	0.133	0.103	0.07	0.118	***	0.11	0.1	0.117	0.091	0.091	0.102	0.189	0.115	0.108	0.095	0.119	0.095	0.096	0.128
MALL	**0.136**	0.088	0.093	0.148	0.104	0.091	0.093	0.111	0.089	0.12	0.099	0.09	0.137	0.118	***	0.059	0.06	0.055	0.044	0.085	0.177	0.098	0.105	0.063	0.11	0.081	0.095	0.132
MEN	**0.098**	0.066	0.073	0.15	0.079	0.098	0.065	0.052	0.064	0.126	0.073	0.068	0.11	0.108	0.062	***	0.051	0.032	0.033	0.07	0.135	0.077	0.085	0.047	0.108	0.071	0.091	0.098
ORM	**0.122**	0.094	0.085	0.16	0.112	0.097	0.092	0.093	0.08	0.135	0.079	0.075	0.125	0.126	0.063	0.054	***	0.062	0.058	0.074	0.146	0.094	0.084	0.058	0.125	0.068	0.109	0.131
CHU	**0.109**	0.074	0.069	0.144	0.074	0.075	0.058	0.062	0.072	0.116	0.076	0.064	0.109	0.098	0.059	0.034	0.066	***	0.028	0.052	0.127	0.073	0.076	0.056	0.096	0.063	0.075	0.084
SEG	**0.105**	0.065	0.071	0.14	0.068	0.07	0.046	0.06	0.068	0.115	0.069	0.06	0.093	0.097	0.046	0.034	0.061	0.03	***	0.054	0.128	0.073	0.065	0.037	0.091	0.064	0.069	0.086

CAN	**0.143**	0.114	0.101	0.144	0.103	0.087	0.073	0.087	0.1	0.139	0.093	0.081	0.134	0.11	0.09	0.074	0.079	0.056	0.057	***	0.11	0.092	0.091	0.089	0.113	0.082	0.106	0.116
PAL	**0.248**	0.187	0.169	0.269	0.162	0.186	0.159	0.15	0.189	0.238	1.157	0.163	0.208	0.21	0.196	0.146	0.159	0.137	0.137	0.117	***	0.154	0.178	0.161	0.191	0.166	0.175	0.196
PB	**0.122**	0.111	0.101	0.2	0.062	0.118	0.098	0.103	0.096	0.141	0.085	0.086	0.128	0.124	0.104	0.082	0.101	0.077	0.077	0.098	0.168	***	0.11	0.086	0.123	0.076	0.088	0.13
ME	**0.121**	0.095	0.103	0.131	0.098	0.09	0.083	0.11	0.085	0.148	0.11	0.105	0.153	0.117	0.113	0.091	0.089	0.081	0.068	0.096	0.197	0.118	***	0.064	0.136	0.108	0.118	0.123
PCHI	**0.115**	0.075	0.083	0.136	0.075	0.087	0.071	0.08	0.069	0.13	0.083	0.078	0.121	0.101	0.066	0.05	0.061	0.059	0.038	0.094	0.176	0.091	0.068	***	0.116	0.087	0.088	0.113
BAL	**0.194**	0.154	0.122	0.211	0.112	0.0154	0.125	0.158	0.113	0.14	0.089	0.115	0.147	0.128	0.118	0.116	0.135	0.103	0.097	0.121	0.212	0.132	0.148	0.124	***	0.062	0.047	0.124
SI	**0.152**	0.104	0.088	0.17	0.079	0.099	0.086	0.121	0.078	0.117	0.07	0.075	0.117	0.102	0.086	0.076	0.072	0.067	0.067	0.087	0.183	0.08	0.116	0.092	0.065	***	0.048	0.125
UDA	**0.163**	0.099	0.106	0.197	0.076	0.107	0.1	0.122	0.087	0.147	0.091	0.092	0.125	0.103	0.101	0.097	0.117	0.08	0.072	0.113	0.194	0.094	0.127	0.093	0.049	0.051	***	0.103
DORP	**0.198**	0.157	0.172	0.185	0.099	0.146	0.119	0.135	0.109	0.197	0.134	0.094	0.138	0.147	0.153	0.118	0.154	0.102	0.101	0.135	0.223	0.153	0.143	0.13	0.143	0.145	0.123	***
